# Structural determinants of M2R involved in inhibition by Sigma-1R

**DOI:** 10.1016/j.jbc.2024.108006

**Published:** 2024-11-17

**Authors:** Chang Liu, I-Shan Chen, Muruj Barri, Ruth Murrell-Lagnado, Yoshihiro Kubo

**Affiliations:** 1 Division of Biophysics and Neurobiology, Department of Molecular and Cellular Physiology, National Institute for Physiological Sciences, National Institutes of Natural Sciences, Okazaki, Japan; 2 Program of Physiological Sciences, Field of Life Science, Department of Advanced Studies, SOKENDAI (The Graduate University for Advanced Studies), Hayama, Japan; 3 Department of Pharmacology, Faculty of Medicine, Wakayama Medical University, Wakayama, Japan; 4 School of Life Sciences, University of Sussex, Brighton, UK

**Keywords:** M2-muscarinic acetylcholine receptor, sigma-1 receptor, inhibition, structural determinants, patch-clamp

## Abstract

Sigma-1 receptor (S1R) is a multimodal chaperone protein that is implicated in various pathophysiological conditions including drug addiction, Alzheimer’s disease, and amyotrophic lateral sclerosis (ALS). S1R interacts with various ion channels and receptors on the endoplasmic reticulum or plasma membrane (PM). It has been reported that S1R colocalizes with the M2-muscarinic acetylcholine receptor (M2R) on the soma of motoneurons, although a functional interaction between these two proteins has not been established. Here, we investigated the regulation of M2R signaling by S1R using electrophysiological recordings of GIRK currents in HEK293T cells. We observed that S1R strongly inhibited M2R-mediated activation of GIRK1/2, but the disease mutant linked to ALS, S1R E102Q, did not. The inhibitory effect of S1R was selective for M2R and wasn’t seen when S1R was co-expressed with other G_i/o_ coupled receptors including M4R. Chimeric and mutant receptors of M2R and M4R were generated and analyzed, and this highlighted Ala401 in the transmembrane 6 domain (TM6) of M2R and Glu172 as well as Glu175 in the extracellular loop 2 regions of M2R, as essential for the inhibition by S1R. Co-immunoprecipitation confirmed the physical interaction between M2R and S1R. Immunocytochemical labeling of M2R and S1R expressed in HeLa cells, HEK293T cells, and cultured hippocampal neurons, showed clear PM expression of M2R throughout the cell which was decreased by coexpression with S1R but was still apparent. Taken together, our results show that S1R interacts with M2R to reduce both its PM expression and function, and this involves TM6 and the extracellular loop 2.

The Sigma-1 receptor (S1R) is widely expressed in the brain and in the periphery, acts as a chaperone protein, and is a regulator of ion channels and receptors (1, 2). It has been extensively studied in physiological and pathophysiological processes in the central nervous system including pain, memory, Parkinson’s disease, Alzheimer’s disease, and also juvenile amyotrophic lateral sclerosis (ALS). There is a well-known ALS-linked disease mutant of S1R, E102Q, which causes ER defects and proteotoxic stress leading to the aggregation of ALS-linked RNA-binding proteins and subsequently resulting in the occurrence of the ALS pathogenesis in motor neurons (3).

Different oligomerization states from monomer to octamer of S1R have been reported (4, 5), with agonists of S1R suggested to stabilize S1R in its monomer and dimer forms while antagonists disrupted S1R function by keeping it in its higher oligomer forms (1). S1R was originally proposed to have two hydrophobic helical transmembrane domains (TMs) by the sequence-based algorithms including amino acid hydrophobicity analyses and structural flexibility assessment plots (1). Based on this two-TMs model, both N- and C-terminal of this receptor were assumed to be located in the lumen (extracellular) side of ER. In contrast to the 2 TMs model of S1R, the first crystal structure of human S1R, however, revealed a trimeric architecture with only 1 TM in each protomer and suggested that the N-terminus (N-ter) is located on the luminal side of the ER while the C-terminus (C-ter) is located on the cytosolic side (6). Recently, further investigation of the topology of S1R was carried out using a glycosylation mapping approach, and this indicated that S1R is a type II, ER-localized membrane protein whose C-ter is located on the luminal side of the membrane (7).

The C-ter of S1R is referred to as the ligand binding site and contains steroid-binding-like domains (SBLD) I and II (8, 9, 10). The receptor is regulated by a diverse array of ligands that bind with high affinity at the SBLD I and SBLD II regions, including drugs of abuse and those in clinics used to treat depression. Drugs such as pentazocine, cocaine, and fluvoxamine are classified as agonists of S1R, whereas progesterone and haloperidol are classified as antagonists. An agonist and antagonist used to study S1R function experimentally are (+)-N-Allylnormetazocine hydrochloride (SKF10047) and BD1047 dihydrobromide (BD1047), respectively (11). In addition, progesterone has been suggested to be an endogenous ligand of S1R involved in reducing capsaicin-induced pain by impairing an interaction between S1R and the TRPV1 channel (12).

At rest, S1R is located at the mitochondrion-associated ER membrane (MAM) region and the nuclear envelope. At the MAM, it plays an important role in chaperoning the IP_3_ receptor (IP_3_R) to maintain the Ca^2+^ homeostasis between the ER and mitochondria (13). In response to cellular stress or in the presence of agonists, S1R can dynamically redistribute towards the PM, although it remains unclear as to whether it remains within the ER at ER-PM junctions or can insert into the PM (2, 11, 14, 15, 16, 17). S1R has been shown to directly/indirectly regulate the function of a variety of membrane proteins including voltage-gated ion channels, G protein-coupled receptors, the N-methyl-D-aspartate receptor (NMDAR), and stromal interaction molecule 1 (STIM1) (2, 11, 18, 19). For example, S1R was shown to diminish the current amplitude and accelerate the inactivation of K_v_1.4 channels, an effect that was enhanced by its agonist SKF10047 (20). The mechanisms involved in S1R regulation of PM proteins are still unclear, for example, does S1R remain within the ER at the ER-PM junctions or translocate to the PM, and what are the structural determinants involved in the interactions?

Muscarinic acetylcholine M2 receptors (M2R) are G_i/o_ coupled receptors important in regulating the electrical activity of both neurons and cardiac myocytes. S1R has been reported to colocalize with M2R on the soma of motoneurons in the mouse brainstem and spinal cord by immunohistochemical staining (IHC). Electron microscope images demonstrated that S1R locates mostly on the surface of the ER but not on the PM (11). The close proximity of M2R and S1R suggests that these two proteins might interact at ER-PM junctions. Despite the observation that M2R and S1R colocalize together, a functional interaction between them has not yet been reported.

By electrophysiological recording and IHC using HEK293T cells as an *in vitro* expression system and co-transfection of M2R, G_i/o_-coupled GIRK channel, and S1R, we have identified an interaction between S1R and M2R which regulates both the function and PM expression of the M2R. This regulation was selective for M2R and was not observed for other G_i/o_ coupled receptors. Amino acids within the transmembrane domain (TM) 6 and extracellular loop 2 (ECL2) of M2R were identified as playing an important role in this regulation.

## Results

### Downregulation of M2R signaling by S1R

We examined the effects of S1R on M2R activation in HEK293T cells by coexpressing the G-protein coupled inward rectifier potassium channel half (GIRK1/2). M2R belongs to G_i/o_ coupled receptor family for which GIRK channels are an effector (21, 22). Upon the application of oxotremorine-M (OXO-M), a synthetic agonist of M2R, the current amplitude of GIRK1/2 channels increased as expected, due to the binding of G_βγ_ subunits which are dissociated from G_i/o_ upon activation of M2R (23, 24).

**Figure 1:**
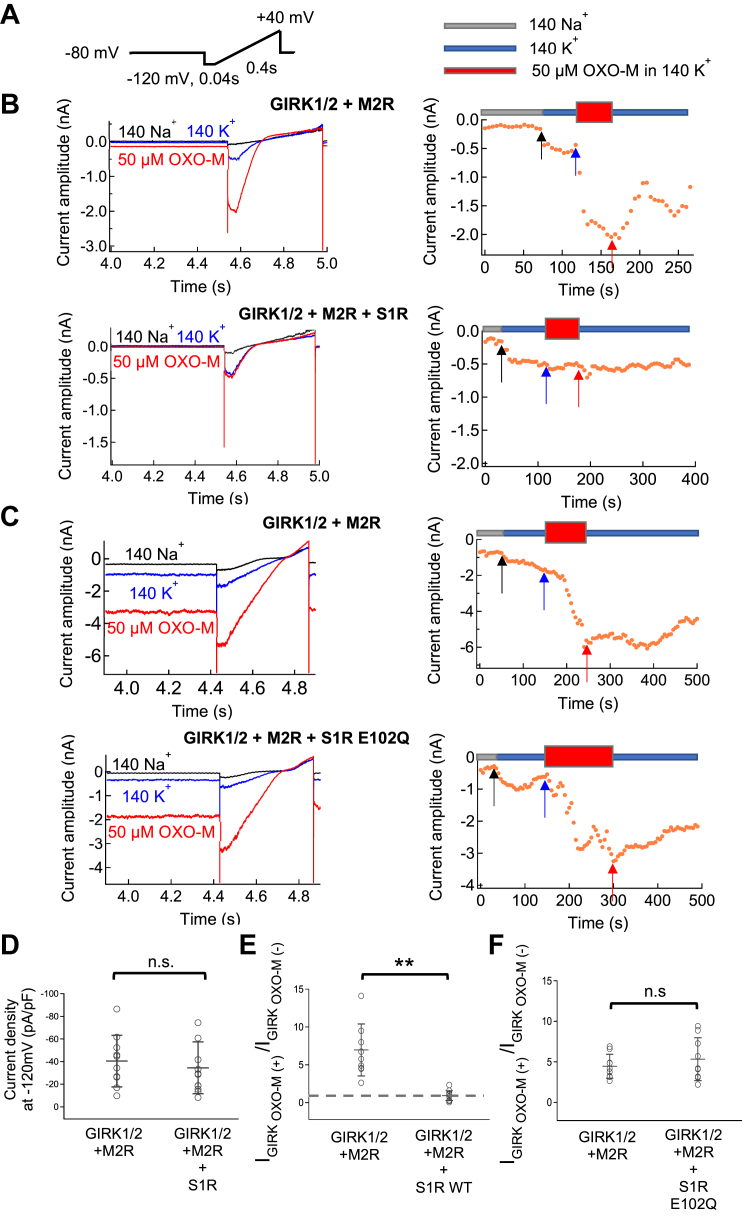
**The regulation of M2R by S1R or S1R E102Q is measured by GIRK1/2 current.***A Left*, A voltage ramp pulse protocol used in whole cell patch-clamp recording. *Right*: Indication of 140Na^+^ (*Gray*), 140K^+^ (*Blue*) or 140K^+^ with 50 μM OXO-M (*Red*) bath solutions which used for recording in bellowing. *B* and *C*, recordings from HEK293T cells transfected with GIRK1/2+M2R, GIRK1/2+M2R+S1R or GIRK1/2+M2R+S1R E102Q. *Left*: Representative current traces evoked by ramp pulses in 140Na^+^ (*Black*), 140K^+^ (*Blue*) and 140K^+^ with 50 μM OXO-M (*Red*), respectively. *Right*: Timelapse changes of the current amplitude at −120 mV in different bath solutions. *Black, blue* and *red arrows* indicate the time points of representative current traces shown in the *left panel* in 140Na^+^, 140K^+^ and 140K^+^ with 50 μM OXO-M, respectively. *D*, comparison of basal GIRK1/2 current densities in GIRK1/2 and M2R transfected HEK293T cells (*left*) or GIRK1/2, M2R and S1R transfected cells (*right*). Data are mean ± SD (n = 10,12). *E*, comparison of the ratio of the GIRK1/2 current before and after application of OXO-M in GIRK1/2+M2R, GIRK1/2+M2R+S1R, transfected cells. Data are mean ± SD (n = 9, 11). *F,* comparison of the ratio of the GIRK1/2 current before and after application of OXO-M in GIRK1/2+M2R, GIRK1/2+M2R+S1R E102Q transfected cells. Data are mean ± SD (n = 9, 9). Student’s *t* test (Unpaired), ∗∗ indicates *p* < 0.01, n.s. indicates no statically significant difference.

When S1R was coexpressed with M2R and GIRK1/2, the OXO-M-induced increase in GIRK currents was inhibited (Fig. 1*B*). The fold increase in the GIRK1/2 current was 7.0 ± 3.4 in the absence of S1R and 0.9 ± 0.6 in its presence (Fig. 1*E*). There was no difference in the basal current amplitude before application of OXO-M between both groups suggesting that S1R did not affect the expression and function of the GIRK1/2 channel itself (Fig. 1*D*) and that the effect was on M2R activation.

We next tested the effect of the ALS-linked S1R E102Q mutant on M2R function. Similar results showing M2R activation were obtained with or without S1R E102Q (Fig. 1*C*). OXO-M induced increase in the GIRK1/2 current was 4.4 ± 1.4 fold in the absence of S1R E102Q and 5.3 ± 2.6 fold in its presence (Fig. 1*F*). This shows that the inhibitory effect of S1R on M2R is lost with the disease-associated S1R E102Q mutant.

### Co-expression of S1R with other G_i/o_ coupled receptors had no effect on downstream activation of GIRK1/2 channel currents

**Figure 2:**
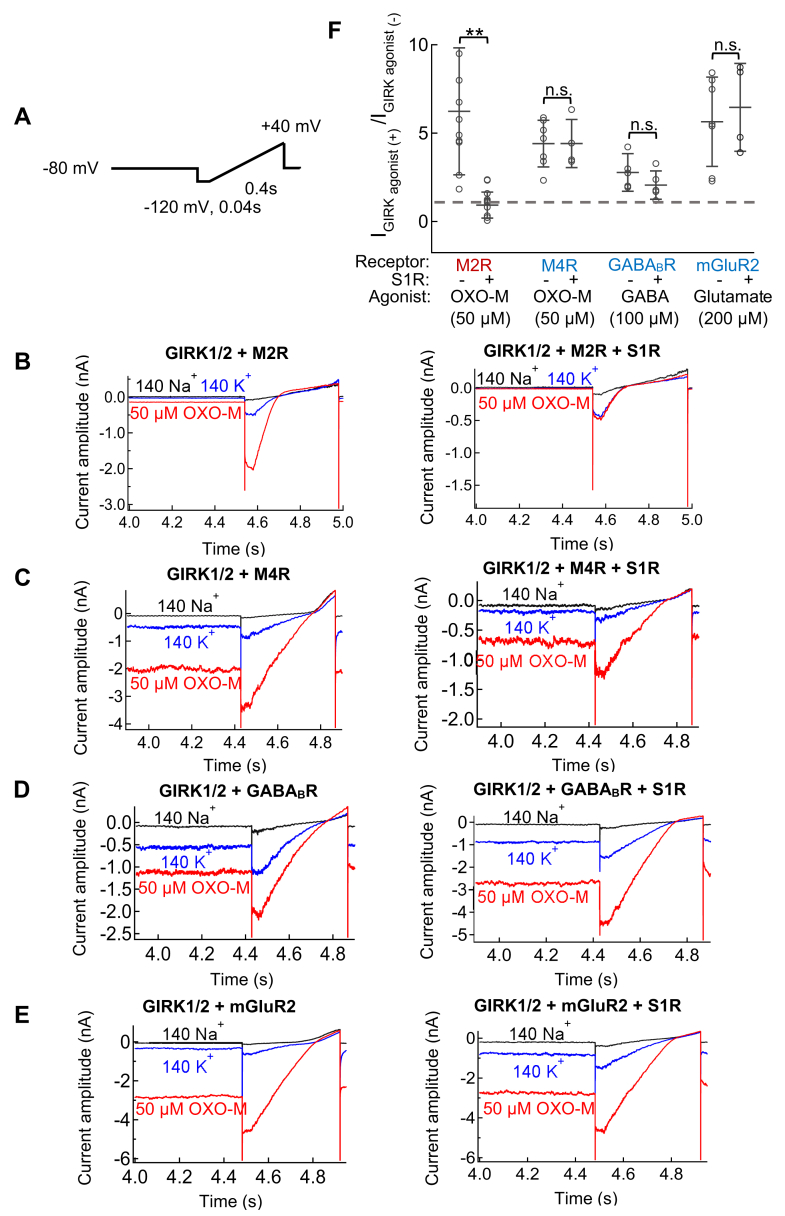
**The regulation of various G**_**i/o**_**coupled receptors by S1R measured by GIRK1/2 current.***A*, a voltage ramp pulse protocol used in whole-cell patch-clamp recording. *B–E*, recordings from HEK293T cells transfected with GIRK1/2+M2R, GIRK1/2+M4R, GIRK1/2+GABA_B_R and GIRK1/2+mGluR2. *Left*: Representative current traces evoked by ramp pulses in the absence of S1R in 140Na^+^ (*Black*), 140K^+^ (*Blue*), and 140K^+^ with 50 μM OXO-M (*Red*), respectively. *Right*: Representative current traces evoked by ramp pulses in the presence of S1R in 140Na^+^ (*Black*), 140K^+^ (*Blue*), and 140K^+^ with 50 μM OXO-M (*Red*), respectively. *F*, comparison of the ratio of the GIRK1/2 current before and after application of their own agonists in GIRK1/2 + M2R, GIRK1/2 + M4R, GIRK1/2+GABA_B_R and GIRK1/2+ mGluR2 without or with S1R co-transfected cells. Data are mean ± SD (n = 6–12). Student’s *t* test 9 (Unpaired), ∗∗ indicates *p* < 0.01.

Other G_i/o_ coupled receptors that share a similar downstream signaling pathway after stimulation by their ligands include the M4-muscarinic acetylcholine receptor (M4R), gamma-aminobutyric acid type B receptor (GABA_B_R), and metabotropic glutamate receptor 2 (mGluR2) (25). We tested the effect of S1R on these receptors by coexpression with GIRK and applying agonists OXO-M, GABA or glutamate. For M4R, OXO-M increased current amplitude by 4.5 ± 1.4 fold without S1R and 3.9 ± 0.8 fold with S1R (Fig. 2, *C* and *F*). For GABA_B_R and mGluR2 the fold increase in GIRK current in the presence of GABA or glutamate was similarly unchanged by co-expression of S1R (for GABA_B_R, 2.7 ± 1.0 *vs.* 2.1 ± 0.7 fold, and for mGluR2, 5.7 ± 2.7 and 6.2 ± 2.2 fold, respectively) (Fig. 2, *D*–*F*). In conclusion, S1R had no inhibitory effect on M4R, GABA_B_R and mGluR2 activation.

In this study, we used porcine M2R, rat M4R, and human S1R, but the amino acid sequences of M2R, M4R, and S1R are very highly conserved in porcine, rat, and human (98.3% for M2R, 97.1% for M4R, 96.4% for S1R among three species) and E102 in S1R is conserved among species. Thus, we trust that our results are not species-specific.

### Regulation of M2R/M4R chimeras by S1R

**Figure 3:**
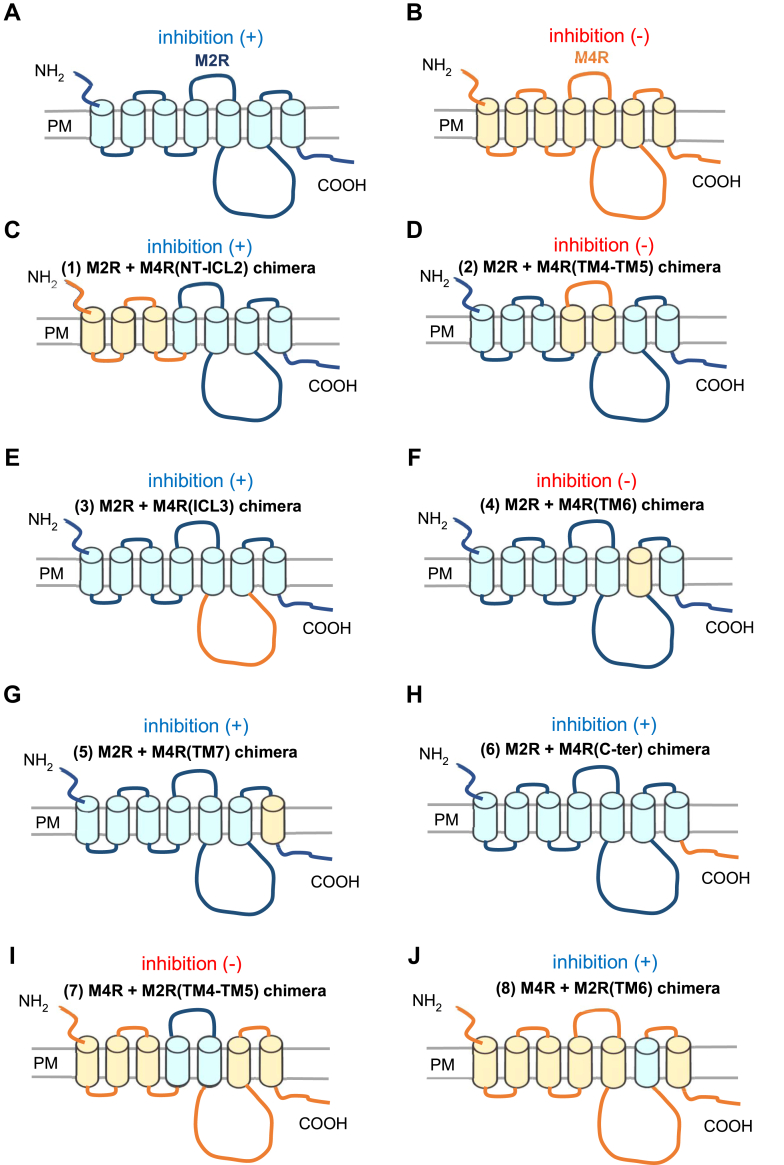
**Schematic drawings of chimeras between M2R and M4R.***A*, the topological model of M2R WT is indicated in *blue*. *B*, the topological model M4R WT is indicated in *yellow*. *Bule* and *yellow* columns embedded in PM indicate TMs from TM1 to TM7 of M2R and M4R respectively. *C–H*, chimeras of M2R (*blue*) in which M4R segments (*yellow*) of NT-ICL2, TM4-TM5, ICL3, TM6, TM7 and C-terminal were introduced. *I* and *J*, Chimeras of M4R (*yellow*) in which M2R segments (*blue*) of TM4-TM5 and TM6 were introduced.

**Figure 4:**
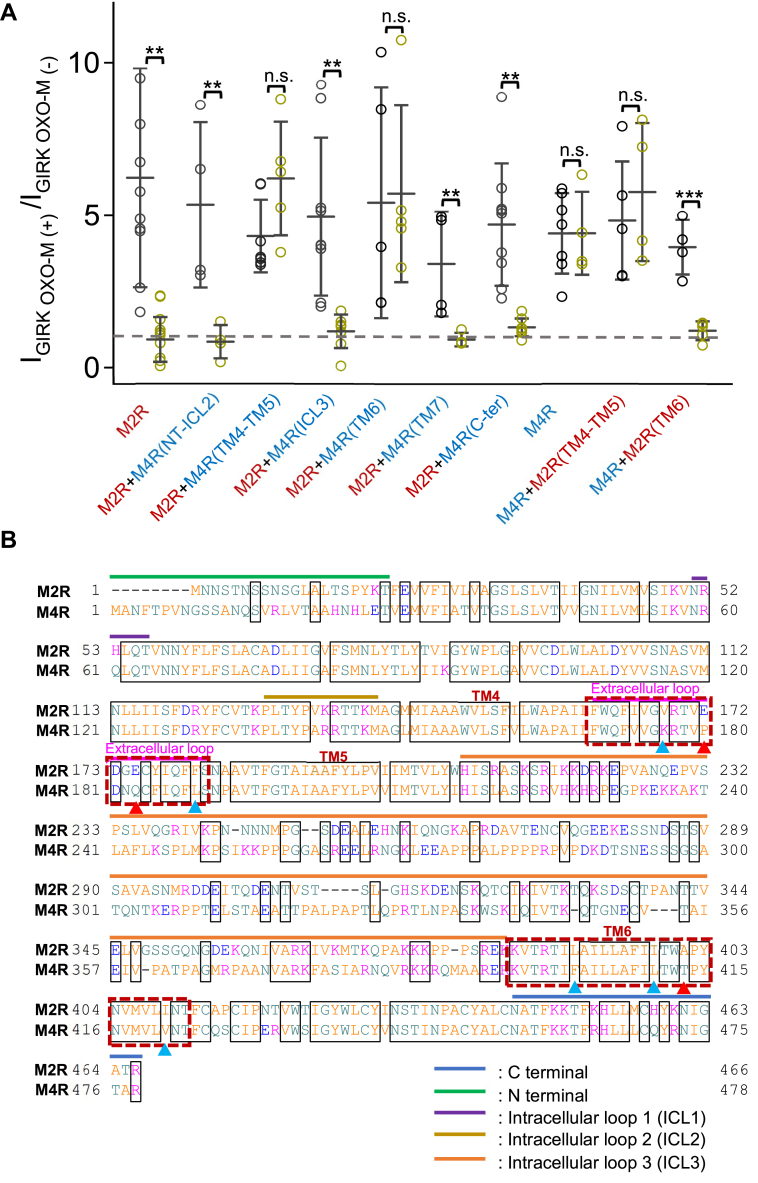
**The regulation of various chimeras between M2R and M4R by S1R measured by GIRK1/2 current.***A*, comparison of the ratio of the GIRK1/2 current before and after application of their own agonists in GIRK1/2+M2R, GIRK1/2+M2R/M4R(NT-ICL2), GIRK1/2+M2R/M4R(TM4-TM5), GIRK1/2+M2R/M4R(ICL3), GIRK1/2+M2R/M4R(TM6), GIRK1/2+M2R/M4R(TM7), GIRK1/2+M2R/M4R(C-ter), M4R, GIRK1/2+M4R/M2R(TM4-TM5), GIRK1/2+M4R/M2R(TM6) without (*black*) or with (atrovirens) S1R co-transfected cells. Data are mean ± SD (n = 4–12). Student’s *t* test (Unpaired), ∗∗ indicates *p* < 0.01, ∗∗∗ indicates *p* < 0.01. *B*, the alignment of the amino acid sequence of M2R and M4R. The full amino acid sequence of porcine M2R and rat M4R are aligned. Red dotted lines indicate TM4 and TM5 of M2R and M4R. Arrows indicate the position of examined single point mutations in the extracellular loop between TM4 and TM5 or in the TM6.

The difference in sensitivity of M2R and M4R to regulation by S1R prompted the use of a chimeric approach to identify the structural determinants involved. Chimeric constructs were generated as shown in Fig. 3. Chimeric receptors in which either the N-terminus to intracellular loop 2 (ICL2) (Fig. 3*C*), the intracellular loop 3 (ICL3) (Fig. 3*E*), the transmembrane domain 7 (TM7) (Fig. 3*G*) or C-terminus (Fig. 3*H*) of M4R were substituted for the analogous regions of M2R retained their ability to activate GIRK currents upon receptor stimulation, and were still inhibited by co-expression of S1R similar to the wild type M2R (Fig. 4*A*). Chimeras in which either transmembrane regions TM4-5 (Fig. 3*D*), or TM6 (Fig. 3*F*) of M2R were substituted for the equivalent regions of M4R (M2R/M4R(TM4-5) and M2R/M4R(TM6)), also retained their ability to activate GIRK currents but lost the inhibitory effect of S1R (Fig. 4*A*). This suggests that TM4-5 and TM6 of M2R are involved in the inhibitory effect of S1R. The reverse chimeras were made, M4R/M2R(TM4-TM5) and M4R/M2R(TM6) (Fig. 3, *I* and *J*), but only M4R/M2R(TM6) acquired the inhibitory effect of S1R (Fig. 4*A*). These data indicate that TM4-TM5 on M2R is important for the inhibition of M2R by S1R, but its presence is not sufficient and that TM6 of M2R is critical for the interaction between M2R and S1R.

### Analysis of point mutations within the TM4-5 and TM6 regions of M2R identify a role for E172, E175, and A401 in S1R regulation

**Figure 5:**
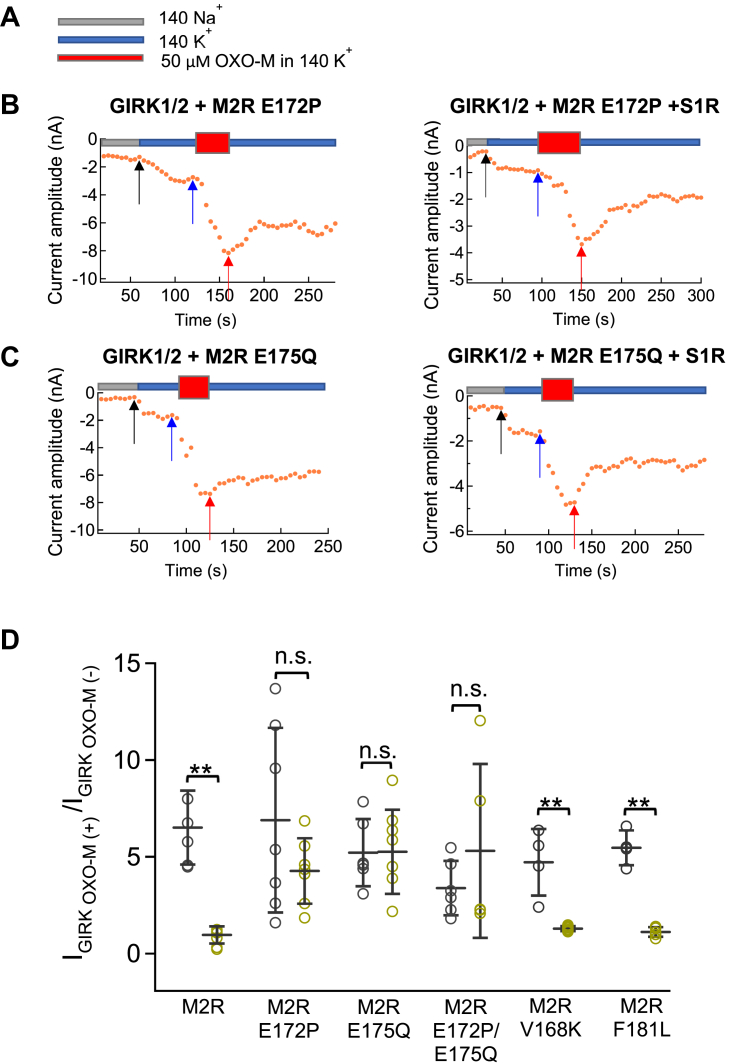
**Regulation on GIRK1/2 current by S1R through M2R and its ECL2 mutants measure by GIRK1/2 current.***A*, indication of 140Na^+^ (*Gray*), 140K^+^ (*Blue*) or 140K^+^ with 50 μM OXO-M (*Red*) bath solutions which used for recording in *B* and *C*. *B* and *C*, timelapse change of the current amplitude at −120 mV in different bath solutions. Recordings from GIRK1/2, M2R E172P and M2R E175Q without (*left*) or with (*Right*) co-transfection of S1R in HEK293T cells. *Black*, *blue* and *red* arrows indicate the time points which picked up for analyzing in 140Na^+^, 140K^+^ and 140K^+^ with 50 μM OXO-M, respectively. *D*, comparison of the ratio of the GIRK1/2 current before and after application of OXO-M in GIRK1/2+M2R, GIRK1/2+M2R E172P±S1R, GIRK1/2+M2R E175Q±S1R, GIRK1/2+M2R E172P/E175Q±S1R, GIRK1/2+M2R V168K±S1R, GIRK1/2+M2R F181L±S1R transfected cells. Data are mean ± SD (n = 4–7). Student’s *t* test (Unpaired), ∗∗ indicates *p* < 0.01.

**Figure 6:**
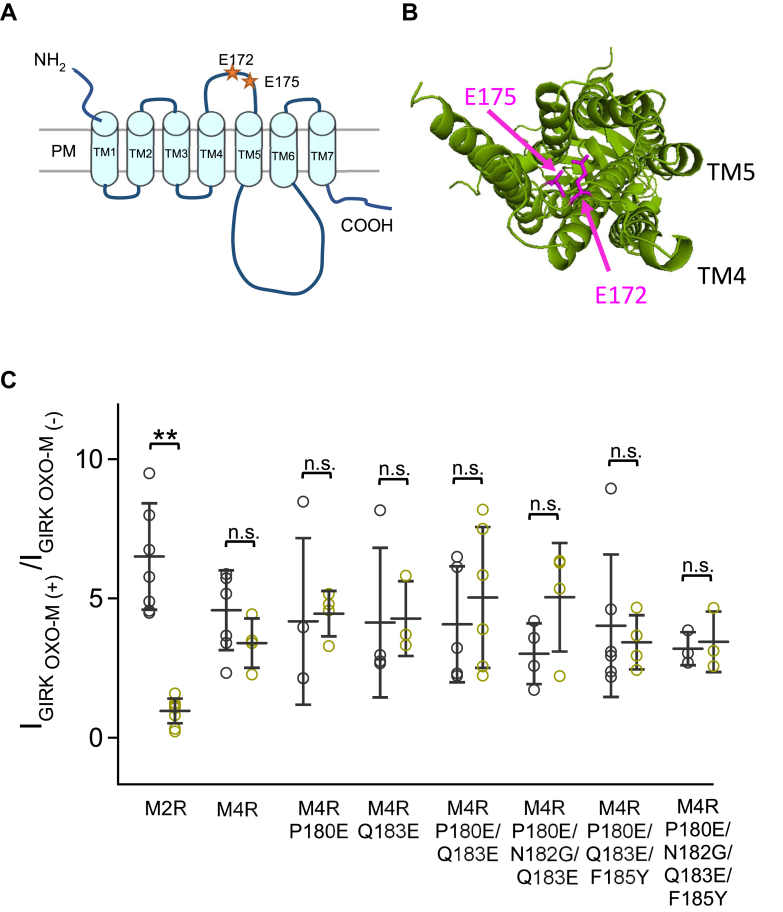
**Regulation of M2R/M4R mutants in TM4-TM6 by S1R.***A*, a schematic view of M2R, *orange asterisks* indicate the location of E172, E175 on M2R. *B*, top view from the extracellular side of the crystal structure of M2R (Human M2R, 4MQS). *Magenta color* depicts the location of E172 and E175 using PyMOL. *C*, comparison of the ratio of the GIRK1/2 current before and after application of OXO-M in GIRK1/2+M2R±S1R, GIRK1/2+M4R±S1R, GIRK1/2+M4R P180E±S1R, GIRK1/2+M4R Q183E±S1R, GIRK1/2+M4R P180E/Q183E±S1R, GIRK1/2+M4R P180E/N182G/Q183E±S1R, GIRK1/2+M4R P180E/Q183E/F185Y±S1R and GIRK1/2+M4R P180E/N182G/Q183E/F185Y±S1R transfected HEK293T cells. Data are mean ± SD (n = 4–7). Student’s *t* test (Unpaired), ∗∗ indicates *p* < 0.01.

To further narrow down the critical region within M2R that is involved in the inhibitory action of S1R, single amino acid substitutions were made based on differences in sequence within the TM4-5 and TM6 regions of M2R compared to M4R (Fig. 4*B*). Within the TM4-TM5 region, single point mutants of Val168, Glu172, Glu175, and Phe181 of M2R to the corresponding amino acid Lys, Pro, Gln, and Leu of M4R (M2R V168K, M2R E172P, M2R E175Q, M2R F181L) were made (Fig. 4*B*). Only two of these mutant receptors, M2R E172P and M2R E175Q, lacked inhibition by S1R (for M2R E172P, activation ratios for OXO-M were 6.9 ± 4.8-fold in the absence of S1R and 4.3 ± 1.7 fold in its presence and for M2R E175Q, activation ratios were 5.2 ± 1.7 fold in the absence of S1R, and 5.3 ± 2.2 fold in its presence) (Fig. 5, *B*–*D*). The double mutant of M2R, E172P/E175Q, also showed no inhibition by S1R (Fig. 5*D*). These results suggest that Glu172 and Glu175 on the extracellular loop of M2R (Fig. 6, *A* and *B*) between TM4 and TM5 play critical roles in the inhibition of M2R by S1R.

The reverse mutations were made in M4R, namely P180E, Q183E, and P180E/183E, but these showed no inhibitory effect of S1R (Fig. 6*C*). Further mutations around Glu172 and Glu175 sites including M4R P180E/N182G/Q183E, P180E/Q183E/F185Y, and P180E/N182G/Q183E/F185Y also showed no acquisition of the inhibitory effect of S1R (Fig. 6*C*). The results are consistent with the results of the chimeric approach and show that the TM4-5 region of M2R is not sufficient to confer sensitivity to S1R to the M4R receptor.

**Figure 7:**
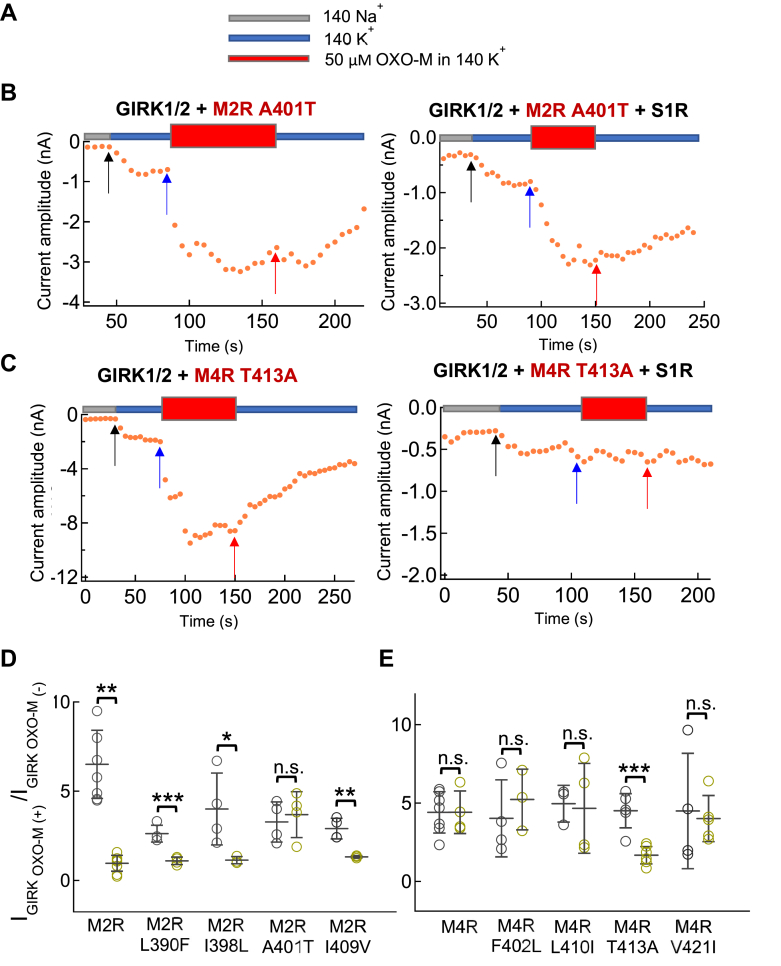
**Regulation on GIRK1/2 current by S1R through M2R and its TM6 mutants measure by GIRK1/2 current.***A*, indication of 140Na^+^ (*Gray*), 140K^+^ (*Blue*) or 140K^+^ with 50 μM OXO-M (*Red*) bath solutions used for recording in *B* and *C*. *B* and *C*, timelapse change of the current amplitude at −120 mV in different bath solutions. Recordings from GIRK1/2, M2R A401T, and M4R T413A without (*left*) or with (*Right*) co-transfection of S1R in HEK293T cells. *Black*, *blue*, and *red arrows* indicate the time points which picked up for analyzing in 140Na^+^, 140K^+^ and 140K^+^ with 50 μM OXO-M, respectively. *D*, comparison of the ratio of the GIRK1/2 current before and after application of OXO-M in GIRK1/2+M2R, GIRK1/2+M2R L390F±S1R, GIRK1/2+M2R I398L±S1R, GIRK1/2+M2R A401T±S1R, GIRK1/2+M2R I409V±S1R transfected cells. *E*, comparison of the ratio of the GIRK1/2 current before and after application of OXO-M in GIRK1/2+M4R, GIRK1/2+M4R F402L±S1R, GIRK1/2+M4R L410I±S1R, GIRK1/2+M4R T413A±S1R, GIRK1/2+M4R V421I±S1R transfected cells. Data are mean ± SD (n = 4–7). Student’s *t* test (Unpaired), ∗ indicates *p* < 0.05, ∗∗ indicates *p* < 0.01, ∗∗∗ indicates *p* < 0.001.

Within the TM6 region, single point mutations were made, individually changing Leu390, Ile398, Ala401 and Ile409 of M2R to the corresponding amino acid within M4R (M2R L390F, M2R I398L, M2R A401T and M2R I409V) (Figs. 4*B*, and 7, *D* and *E*). Only the M2R A401T mutant lost the inhibitory effect of S1R with an activation ratio for OXO-M of 3.3 ± 1.1 fold in the absence of S1R and 3.7 ± 1.3 fold in its presence (Fig. 7, *B* and *D*). Interestingly, the reverse mutation in M4R (M4R T413A) was sufficient to confer inhibition by S1R (Fig. 7, *C* and *E*). The activation ratio for OXO-M was 4.5 ± 1.1 fold in the absence of S1R, and 1.7 ± 0.6 fold in its presence, indicating a key role for the alanine at this position within TM6 of M2R. These amino acids identified as playing a key role in M2R (A401, E172, and E175), are all conserved between porcine, rat, and human M2Rs.

### Co-immunoprecipitation of M2R and S1R suggests an interaction between these two proteins

**Figure 8:**
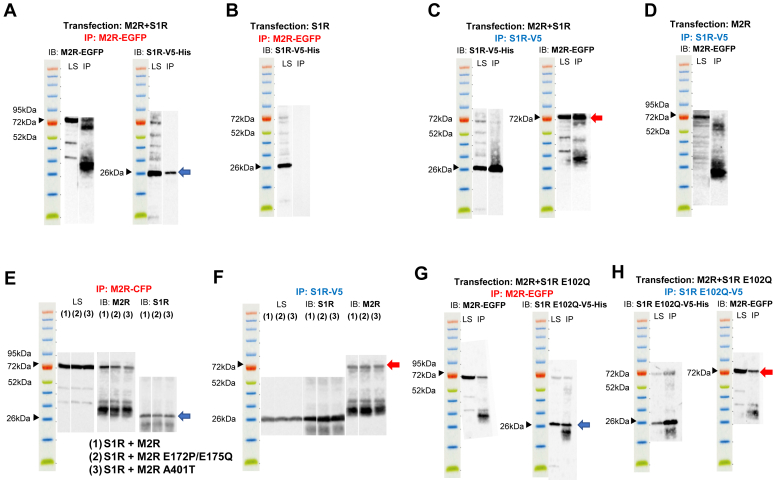
**Co-immunoprecipitation of M2R-EGFP or mutants with S1R-V5-His or S1R E102Q mutant in HEK293T cells.***A* and *G*, S1R or S1R E102Q co-precipitated with M2R. The predicted molecular weights of M2R, S1R (*A*) and S1R E102Q (*G*) including tags are 78.6 kDa, 27.2 kDa and 27.2 kDa respectively. *Left panel*: M2R was precipitated by anti-EGFP tag mouse antibody and detected by anti-M2R rabbit antibody. Cell lysate sample (LS) and IP sample (IP) shows the presence of M2R. *Right panel*: S1R (*A*) or S1R E102Q (*G*) was detected by anti-His tag rabbit antibody. LS shows the expression of S1R (*A*) or S1R E102Q (*G*) and IP shows S1R (*A*) or S1R E102Q (*G*) co-precipitated with M2R (*blue arrow*). *B*, negative control experiments using cells transfected only with S1R and precipitated by anti-EGFP mouse tag antibody (for M2R precipitation). *C* and *H*, M2R co-precipitated with S1R or S1R E102Q. *Left panel*: S1R (*C*) or S1R E102Q (*H*) was precipitated by anti-V5 tag mouse antibody and detected by anti-His tag rabbit antibody. LS and IP samples show the presence of S1R (*C*) or S1R E102Q (*H*). *Right panel*: M2R was detected by an anti-M2R rabbit antibody. LS shows the expression of M2R and the IP sample shows M2R co-precipitated with S1R (*C*) or S1R E102Q (*H*) (*red arrow*). *D*, negative control experiments using cells transfected only with M2R and precipitated by anti-V5 mouse tag antibody (for S1R precipitation). *E* and *F*, co-immunoprecipitation of (1) M2R-CFP+S1R-V5-His (2), M2R E172P/E175Q-CFP+S1R-V5-His and (3) M2R A401-CFP+S1R-V5-His in HEK293T cells. The predicted molecular weights of M2R, M2R E172P/E175Q, M2R A401T and S1R including tags are 78.6 kDa, 78.6 kDa, 78.6 kDa and 27.2 kDa, respectively. *E*, S1R co-precipitated with M2R. M2R was precipitated by an anti-EGFP tag mouse antibody and detected by an anti-M2R rabbit antibody. Cell lysate sample (LS) (*left panel*) and IP sample (IP) (*middle panel*) show the presence of M2R. S1R was detected by an anti-His tag rabbit antibody. IP sample (*right panel*) shows S1R co-precipitated with M2R (*blue arrow*). *F*, M2R co-precipitated with S1R. S1R was precipitated by an anti-V5 tag mouse antibody and detected by an anti-His tag rabbit antibody. LS (*left panel*) and IP sample (*middle panel*) show the presence of S1R. M2R was detected by an anti-EGFP tag rabbit antibody. IP sample (*right panel*) shows M2R co-precipitated with S1R (*red arrow*).

To investigate a possible interaction between M2R and S1R, a co-immunoprecipitation (co-IP) analysis was carried out following their expression in HEK293T cells. M2R tagged with EGFP (M2R-EGFP), and S1R tagged with V5 and His tags (S1R-V5-His) were immunoprecipitated using mouse anti-EGFP and anti-V5 antibodies respectively. In the immunoblot, M2R was detected by an anti-M2R rabbit antibody and S1R was detected by an anti-His rabbit antibody. Cell lysates showed the expression of M2R and S1R with predicted band sizes (78.6 kDa and 27.2 kDa, respectively) (Fig. 8, *A*–*H*). The IP of M2R resulted in the co-IP of S1R (Fig. 8*A*) and similarly, the IP of S1R resulted in the co-IP of M2R (Fig. 8*C*). Negative control experiments showed that bands of molecular mass corresponding to co-precipitated S1R (Fig. 8*B*) or M2R (Fig. 8*D*) were not detected when M2R or S1R were not co-expressed.

Having shown an interaction between the wild-type M2R and S1R, we repeated the experiment with the M2R E172P/E175Q and M2R A401T mutants. Both mutants were shown to co-IP with S1R and *vice versa* and there was no apparent difference in band intensity. The results suggest that the interaction between these two proteins was not clearly compromised by either mutation (Fig. 8, *E* and *F*).

We also performed co-IP analysis of M2R WT and the S1R E102Q mutant and it was observed that the E102 mutant co-IP with S1R and *vice versa* (Fig. 8, *G* and *H*). The presence of a physical interaction as shown by co-IP experiments in M2R E172P/E175Q mutant, M2R A401T mutant (Fig. 8, *E* and *F*), and S1R E102Q mutant (Fig. 8, *G* and *H*), indicates that an interaction between these two proteins is not sufficient to cause an inhibition of M2R signaling by S1R. It suggests that the nature of the interaction between these two proteins is critical for the regulation of M2R function.

### Regulation of PM expression of M2R by S1R

**Figure 9:**
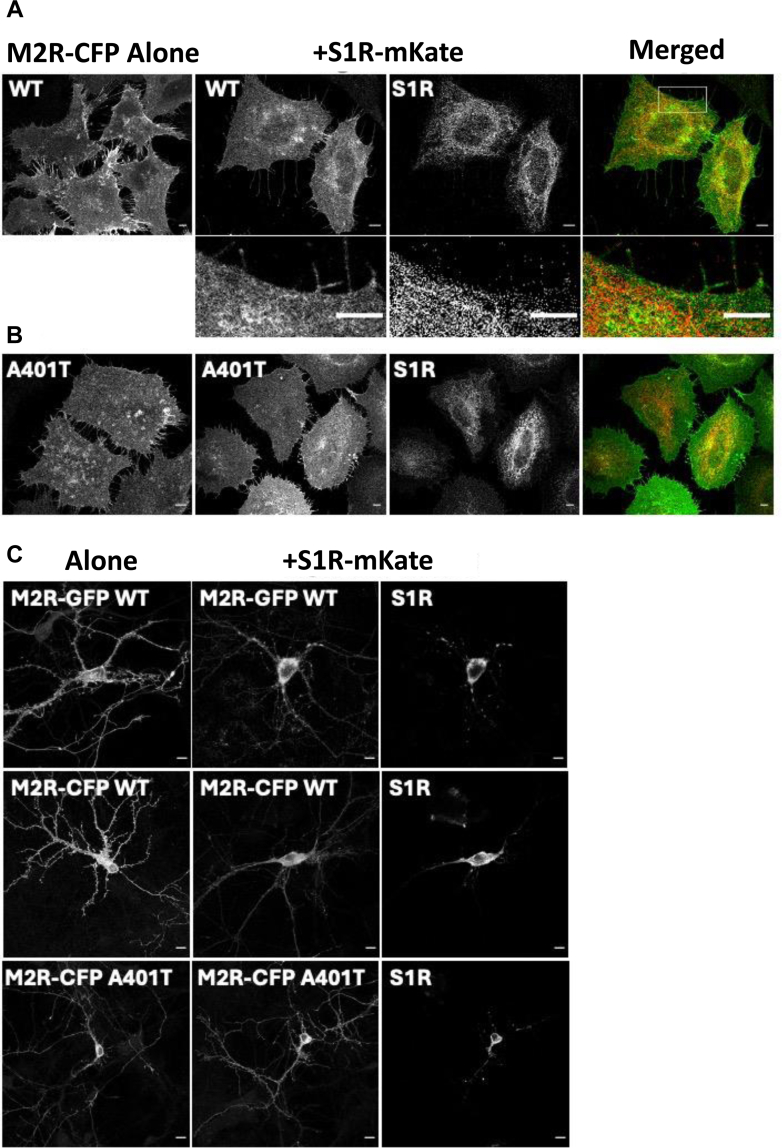
**Impact of S1R on the PM expression of wild type (WT) and A401T mutant M2R.***A*, representative confocal images of HeLa cells expressing either M2R-CFP alone or co-expressed with S1R-mKate. M2R alone was highly expressed at the plasma membrane (PM) as shown by intense labelling of filopodia. When co-expressed it was also at the PM but there was an increase in ER localization. S1R was predominantly intracellular indicating ER localization and there were areas of colocalization with M2R. The zoomed image shows that S1R expression extended towards the cell surface. *B*, images show M2R A401T-CFP expression alone and when coexpressed with S1R-mKate. The mutant receptor was at the PM in both conditions. Scale bars represent 5 μm. *A* and *B*, merged images showed the overlaid fluorescent signals from M2R (*Green*) (*A*) or M2R A401T (*Green*) (*B*) and S1R (*Red*). *C*, *top panel* shows confocal images of live hippocampal neurons expressing M2R-GFP alone and with S1R-mKate. M2R and S1R co-localized at the cell soma and PM expression of M2R within the neurites was reduced in the presence of S1R. *Middle* and *lower panels* show WT M2R-CFP and M2R A401T-CFP respectively, expressed alone or together with S1R-mKate. Neurons were fixed and immunolabelled with an anti-GFP antibody and an AlexaFluor488 conjugated secondary antibody. Alone, M2R showed high expression throughout neurites and within spiney structures and this was reduced by co-expression with S1R. The A401T mutant was expressed throughout neurites with and without S1R but at a reduced level compared to the WT receptor. Scale bars represent 10 μm.

S1R is a chaperone protein known to regulate the PM expression of various ion channels and receptors in addition to regulating their function. We therefore tested its effect on M2R expression using HeLa cells and primary hippocampal neurons. HeLa cells are larger and flatter than HEK293T cells and better suited for imaging the subcellular localization of proteins within cells. Cells expressing M2R-CFP alone were fixed and immunolabelled with anti-GFP antibodies. The confocal images showed clear PM expression of the receptor as indicated by intense labeling of filopodia (Fig. 9*A*). Upon co-expression of M2R-CFP with S1R-mKate, M2R was still clearly expressed at the PM, but there was also increased intracellular expression as indicated by the outline of the nuclei (Fig. 9*A*). S1R-mKate showed a typical intracellular distribution indicating ER localization and in some areas overlapped with M2R expression (Fig. 9*A*). The zoomed image showed the S1R expression extending close to the PM (Fig. 9*A*). The M2R A401T-CFP was expressed at the PM, alone and also in the presence of S1R, although the intensity of expression was lower compared to the WT M2R (Fig. 9*B*).

We also compared the expression of WT and mutant M2Rs in cultured hippocampal neurons because these post-mitotic cells are more physiologically relevant. Imaging of M2R-EGFP and S1R-mKate in live neurons showed extensive expression of M2R throughout neurites when expressed alone, and reduced neurite expression when it was coexpressed with S1R. Both proteins colocalized at the cell soma (Fig. 9*C*). Following the immunolabeling of M2R-CFP receptors in fixed neurons, the high levels of PM expression through the neurites and within spiney structures were apparent (Fig. 9*C*). Again, this was reduced in neurons co-expressing S1R-mKate (Fig. 9*C*). Neurons expressing M2R A401T-CFP both with and without S1R-mKate, showed similar levels of expression throughout neurites although the intensity was reduced compared to WT M2R alone (Fig. 9*C*).

### Qualitative analysis of the effect of S1R WT or E102Q mutant on the PM expression of M2R by S1R

As a reduction of surface expression of M2R when co-expressed with S1R was observed, we next quantified the PM expression of M2R in the absence and presence of S1R. M2R construct with an extracellular HA tag and EGFP at the N-terminal (HA-EGFP-M2R) (26) and mKate tagged S1R or S1R E102Q (S1R-mKate or S1R E102Q-mKate) were used for the experiments. The plasma membrane expression of M2R was imaged by incubating live cells with an HA antibody and by using Alexa Fluor 647 (far red). The fluorescence of EGFP (green) reflects the M2R both on the plasma membrane and in the cell. The expression of S1R WT or E102Q mutant was detected by the fluorescence of mKate tag (red).

**Figure 10:**
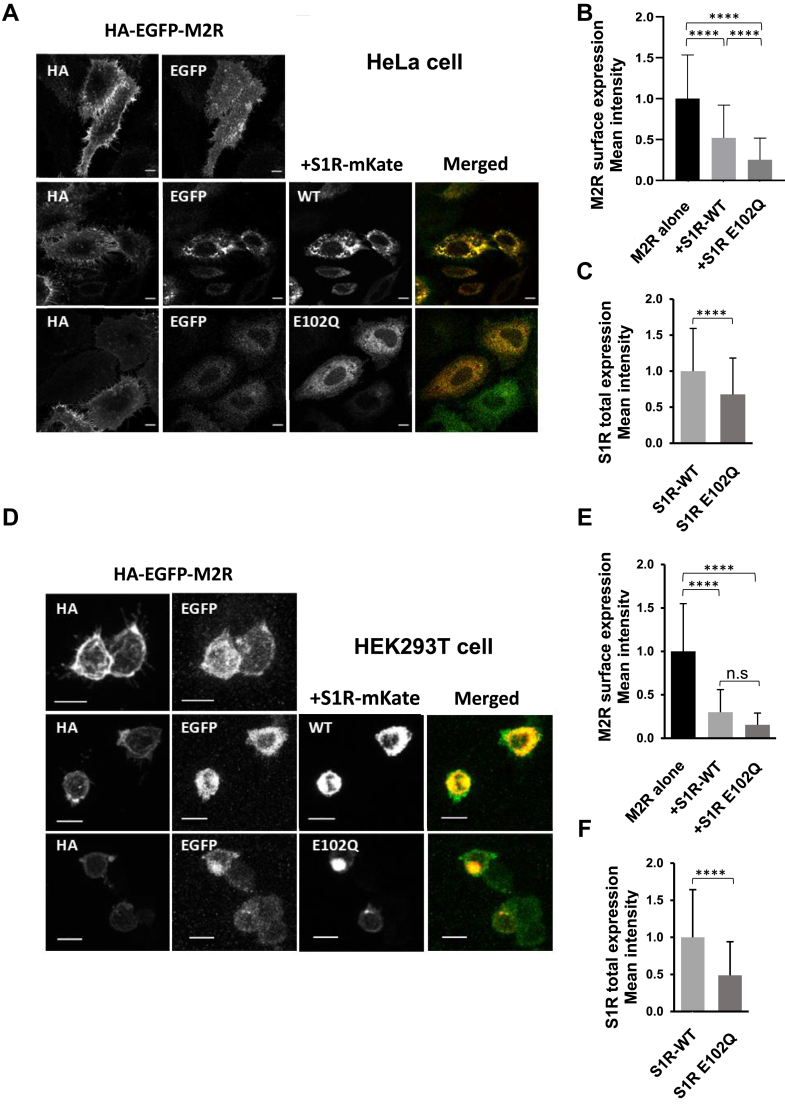
**Quantitative analysis of the effect of co-expression of S1R WT or E102Q mutant on the PM expression of M2R by live cell staining in HeLa cells and HEK293T cells.***A* and *D*, images show HA-EGFP-M2R expression alone (*top panel*) and when coexpressed with S1R-mKate (*middle panel*) or S1R E102Q-mKate (*bottom panel*) in HeLa cells (*A*) or in HEK293T cells (*D*). Scale bars represent 5 μm (*A*) and 10 μm (*D*). *B* and *E*, quantification of surface expression of HA-M2R alone and when coexpressed with S1R-mKate or S1R E102Q-mKate in HeLa cells (*B*) and in HEK293T cells (*E*). Data are presented as mean ± SD for HeLa cells (n = 359, 402, 419 for each group) (*B*) or mean ± SD for HEK293T cells (n = 70, 58, 63 for each group) (*E*). Kruskal-Wallis ANOVA was used to compare the groups in HeLa cells, followed by Dunn’s multiple comparison test (*B*). One-way ANOVA was used to compare the groups in HEK293T cells, followed by Tukey’s analysis for multiple comparisons test (*E*). ∗∗∗∗ indicates *p* < 0.0001, and n.s. indicates no statistically significant difference. *A* and *D*, merged images showed the overlaid fluorescent signals from M2R-EGFP (*Green*) and S1R-mKate (*Red*) or S1R E102Q-mKate (*Red*). *C* and *F*, quantification of the total expression of S1R WT or E102Q mutant by mKate fluorescence intensity when coexpressed with M2R in HeLa cells (*C*) and in HEK293T cells (*F*). Data are presented as mean ± SD for HeLa cells (n = 402, 416 for each group) (*C*) and for HEK293T cells (n = 63, 58 for each group) (*F*). Student’s *t* test was used for statistical analysis. ∗∗∗∗ indicates *p* < 0.0001.

It was observed in HeLa cells that the co-expression of S1R WT significantly reduced the surface expression of M2R to 52.1 ± 40.1% compared to M2R alone (Fig. 10, *A* and *B*) and there was a further decrease of PM expression of M2R in the presence of S1R E102Q to 25.2 ± 26.6% (Fig. 10, *A* and *B*). Similar results were obtained in HEK293T cells which we used for electrophysiological analysis, a decrease to 30.0 ± 26.0% by S1R, and a decrease to 15.5 ± 13.5% by S1R E102Q mutant (Fig. 10, *D* and *E*).

We also compared the total amount of expressed S1R WT and E102Q mutant by the mKate fluorescence intensity when coexpressed with M2R. In HeLa cells, the values were 100.0 ± 59.3% for S1R WT, and 67.8 ± 50.4% for E102Q mutant (Fig. 10*C*). Also, in HEK293T cells, they were 100.0 ± 64.3% for S1R WT and 48.8 ± 45.3% for E102Q mutant (Fig. 10*F*).

## Discussion

This study aimed to characterize the regulation of M2R by S1R and to identify the structural determinants involved in this process. By using a whole cell patch clamp to measure GIRK1/2 currents activated downstream of M2R stimulation, we have demonstrated that the S1R selectively inhibits M2R signaling while not affecting the activation of GIRK1/2 currents mediated by other G_i/o_ coupled receptors. Mutational analysis identified Glu172 and Glu175 within the ECL2 region and Ala401 within the TM6 of M2R as playing an important role in the inhibition of M2R by S1R. Co-immunoprecipitation results indicate that M2R and S1R form a complex, by either a direct or indirect interaction between the two proteins. This reduces PM expression of M2R, but this does not appear to be the primary mechanism by which S1R inhibits M2R function.

### Regulation of M2R by S1R and its mutant S1R E102Q

Since M2R is highly expressed in cholinergic neurons in the brain (27, 28) and the chaperone protein S1R is also expressed throughout both the central and peripheral nervous system (29), it is possible that there might be an interaction between M2R and S1R. It was confirmed that M2R and S1R are co-localized in the postsynaptic side on the soma of motoneurons in the mouse brainstem and spinal cord (11).

In the present study, we showed that S1R inhibits the function of M2R but the ALS-linked mutation, S1R E102Q did not (Fig. 1). What underlies this difference remains to be established. Although the expression of the E102Q mutant was reduced compared to WT S1R it was still able to interact with M2R (Fig. 8*G*) and it produced an even greater reduction in the plasma membrane expression of M2R compared to the WT S1R (Fig. 10, *B* and *E*). Our finding that a ∼85% reduction in M2R surface expression in HEK293T cells in the presence of S1R E102Q was not sufficient to impair M2R-dependent activation of GIRK currents strongly suggests that under the conditions of our experiments, there were spare M2Rs. Based upon these observations, we conclude that the inhibition of M2R-mediated activation of GIRK currents by S1R involved an inhibition of M2R function. The nature of the interaction between S1R and M2R that is involved in this regulation is altered by the S1R E102Q mutation such that the regulation of M2R signaling is lost. It is also notable that the total expression of S1R E102Q was significantly lower than that of S1R WT in both HeLa cells (Fig. 10*C*) and HEK293T cells (Fig. 10*F*). It is possible that this decrease could, at least partly, contribute to the absence of an inhibitory effect by the E102Q mutant.

In previous reports (30, 31), it was discussed that the E102Q mutation destabilizes the interaction between the bulky C-ter and the N-ter transmembrane helix and consequently changes the overall structure of S1R (30, 31). It is therefore to be expected that this would produce changes to the normal trafficking of S1R and the way it interacts with target proteins.

### The mechanism of the interaction between M2R and S1R

Accumulated studies over more than 2 decades have demonstrated the direct/indirect interaction of S1R with a variety of proteins including ionotropic receptors (IP_3_R, NMDAR), voltage-gated ion channels (K^+^, Na^+^, Ca^2+^, K_ir_), thermosensitive ion channel (TRPV1, TRPA1), and G-protein coupled receptors (D1R, D2R) in various subcellular compartments of the cells (11, 12, 18, 32, 33). Surprisingly, the nature of this interaction remains unclear.

It was reported that an interaction between TRPV1 and S1R modulates capsaicin-induced pain. This interaction was demonstrated by co-immunoprecipitation and TM1-TM6 of TRPV1 was shown to be involved in the interaction with S1R (12). S1R colocalized with TRPV1 within the ER, but not at the PM, suggesting that either S1R acts to regulate the stability and trafficking of TRPV1 to the PM and/or it interacts with PM TRPV1 at ER-PM junctions.

In the present study, we showed the involvement of TM6 of M2R by chimera studies between M2R and M4R (Fig. 4) and identified the critical site, Ala401 on TM6 (Fig. 7). In addition to Ala401, we also identified Glu172 and Glu175 on the second extracellular loop between TM4 and TM5 as critical sites for the inhibition of M2R by S1R (Fig. 5). The confocal images of HeLa cells, HEK293T cells, and hippocampal neurons showed a reduction in PM expression of M2R by S1R, but the resolution was not sufficient to identify a possible interaction occurring at ER-PM junctions.

**Figure 11:**
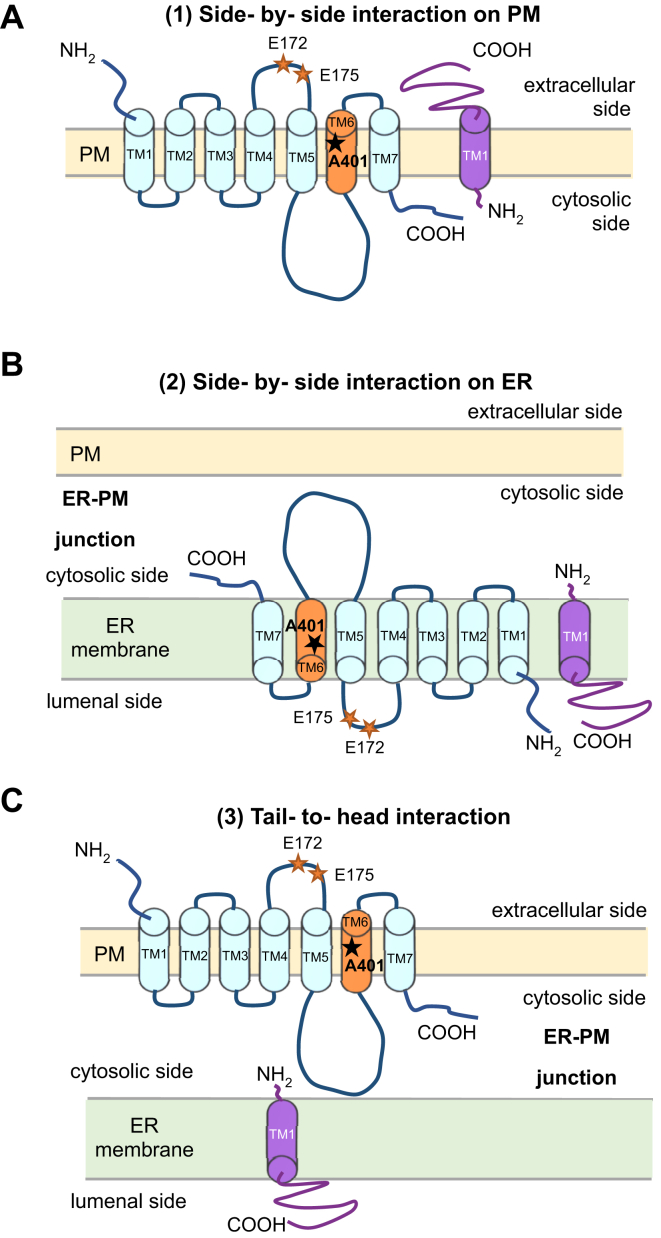
**Schematic drawings of the interaction manners between M2R and S1R.***A*, side-by-side model on PM: M2R and S1R both locate on PM membrane and they interact with each other. *B*, side-by-side model on ER: M2R and S1R both locate on ER membrane and they interact with each other. *C*, tail-to-head model: M2R locates on PM and S1R locates on ER and they interact with each other at the ER-PM junction. In these schemes, the topology of S1R is based on the recent report (7).

Three interaction models could be proposed. They are side-by-side interaction models on the PM (Fig. 11*A*) or within the ER membrane (Fig. 11*B*) *versus* a head-to-tail interaction at the ER-PM junction (Fig. 11*C*). These schemes are drawn based on the recently reported modified topology of S1R with the C-ter tail located not on the cytoplasmic but on the luminal (extracellular) side (7).

Model (2) (Fig. 11*B*) explains the retention of M2R by S1R within the ER (Fig. 9), however, there still remained significant expression of M2R on the PM in HeLa cells, HEK293T cells, and hippocampal neurons in the presence of S1R (Fig. 9 and 10). Thus, the loss of M2R signaling in HEK293T cells cannot be fully explained by its downregulation at the PM in Model (2), suggesting that there is also an inhibition of PM M2R function by a direct molecular interaction with S1R.

As to the functional inhibition of M2R on PM, which case of Model (1) or (3) is more likely? S1R is mostly expressed on ER and the expression in the proximity of PM, presumably at the ER-PM junction, was observed (11) (Fig. 9). Considering this subcellular localization of S1R, Model (3), which depicts the tail-to-head interaction, is more likely. However, only the very short N-ter of S1R is extended to the cytoplasmic region in the ER-PM junction. Thus, the role of A401 in TM 6, and especially E172 and E175 in the extracellular loop of M2R, in its regulation by S1R remains unclear. One possible explanation for the involvement of Glu172 and Glu175 is that they are allosteric sites of M2R (18), and that mutation at these positions might result in the conformational change of M2R. It will lead to the change of other interaction site(s) on M2R such as the third cytoplasmic loop, which results in the loss of the functional interaction between M2R and S1R. Also, there may remain a possibility that the C-ter of S1R is located on the cytoplasmic side, as previously reported by the structure analysis (6).

Model (1), side-by-side interaction on PM, explains well the involvement of TM6 and the extracellular loop of M2R in the functional inhibition by S1R, as the long C-ter of S1R is located on the extracellular side and the expression of S1R on PM was previously reported (14, 15). The expression of S1R extending to the proximity of PM (Fig. 9) might also indicate the expression of S1R on PM, although it could not be explicitly concluded by the spatial resolution of the confocal imaging in the present study. It may not be surprising that M2R and S1R form a molecular complex on ER and are co-transported to the PM.

To further examine the validity of these models and understand the molecular mechanisms involved in S1R-mediated inhibition of M2R, a higher resolution structural analysis of the M2R/S1R complex is required, for example in lipid nano disc(s) by single particle structure analysis using cryo-EM.

### Physiological implications of the functional interaction between M2R and S1R

The inhibition of M2R function and expression by S1R might have some physiological implications, especially in the nervous system where M2R and S1R are both expressed. M2R plays a significant role in cholinergic signaling in the brain, influencing cognition, memory, and neuroplasticity (34, 35). We observed a decrease in the expression of M2R on the PM and more importantly a functional inhibition of M2R activation by recording GIRK current. By inhibiting M2R expression and function, S1R might regulate acetylcholine-mediated signaling, to a suitable level to achieve proper cognitive functions such as learning and memory. Improper regulation of M2R by S1R, especially by S1R mutants, might cause abnormalities such as Alzheimer’s disease, where S1R and cholinergic downstream signaling have been reported to be involved (1, 2, 35).

M2R is a G-protein coupled receptor that primarily mediates parasympathetic nervous system activity, particularly in slowing heart rate (36, 37). It was reported that S1R knockout mice associated with cardiac dysfunction (38) and its ligands such as (+)-pentazocine which is known as an agonist of S1R could cause the changes in heart beating frequencies by modulation of Ca^2+^ influx (39). It is possible that S1R could regulate heartbeat by inhibiting M2R, in addition to the effect on the Ca^2+^ dynamics.

## Experimental procedures

### Ethical approval of animal experiments

C57Bl/6J mice were maintained under conventional housing conditions and a 12–12 light-dark cycle. Neonatal mice (postnatal age 0–2 days) used for experiments were culled by an appropriate Schedule 1 procedure (cervical dislocation) in accordance with the Animals Scientific Procedures Act 1986 amendment regulations 2012. Schedule 1 training of the experimenters was overseen and approved by the local Named Training and Competency Officer (NTCO). Experiments using mice were reviewed *a priori* and approved by the Animal Welfare Ethical Review Body at the University of Sussex.

### Culture and transfection of mammalian cell lines

Human embryonic kidney 293T (HEK293T) cells were cultured in DMEM medium with L-Glutamine and Phenol Red (FUJIFILM Wako Pure Chemical Corporation), supplemented 10% fetal bovine serum (FBS) (ICN Biomedicals, Inc), and 1% penicillin/streptomycin (P/S) (FUJIFILM Wako Pure Chemical Corporation) and maintained in a 5% CO_2_ atmosphere at 37 °C. Transfection of HEK293T cells for patch clamp experiments with cDNAs encoding GIRK1/2 (1 μg), M2R (1 μg), and S1R (1 μg) was carried out using Lipofectamine 2000 reagent (Invitrogen) following the protocol by suppliers for patch-clamp experiments and immunohistochemical staining. After 36 to 48 h, cells were re-seeded onto Poly-L-Lysine (PLL) coated glasses for patch-clamp recording. For co-immunoprecipitation experiments, cDNAs encoding M2R (2 μg) and S1R (2 μg) were transfected using Avalanche-Everyday Transfection Reagent (Avalanche- Everyday) following the protocol by suppliers.

HeLa cells, derived from human cervical carcinoma, were cultured in DMEM/F12 (1:1) medium (Gibco, 21,331–020), supplemented with 1% GlutaMax (Gibco, 35050-038), 10% fetal bovine serum (FBS) (Merck, F9665), and 1% penicillin/streptomycin (P/S) (Gibco, 15140122) and maintained in a 5% CO_2_ atmosphere at 37 °C. For experiments involving immunofluorescence and confocal imaging, cells were seeded onto 16 mm collagen-coated coverslips (Cell, 125–50) and transfected 24 h after plating when they reached a confluency of 40 to 60%. The transfection reagent Transit-LT1 (Mirus, MIR 2304) was used to transfect HeLa cells with 1 μg of M2R alone or in combination with 0.2 μg of Sigma1R. Experiments were performed 48 h post-transfection.

### Culture and transfection of primary hippocampal neurons

To establish hippocampal neuron cultures, three to 4 mouse C57BL/6 pups of either sex, aged between postnatal Days 1 and 2, were utilized to seed six wells of a 12-well plate. Briefly, the hippocampus was dissected in 1× Hank’s Balanced Salt Solution (HBSS, 14155048) supplemented with 1% P/S and dissociated in 2 ml of a plating medium by gently pipetting up and down until a uniform mixture was optioned. The plating media comprises Minimum Essential Media (MEM) (Gibco, 51200087) supplemented with 20 mM D-(+)-Glucose (Marck, 24895335), 1% P/S, 1% sodium pyruvate, HEPES (Merck, H0887), 1× N2 (Invitrogen, 17502048), and 10% heat-inactivated horse serum (Gibco, 26050-088). The mixture was further diluted with the plating media before applying 0.5 ml onto 16 mm coverslips pre-coated with poly-D-lysine (50 μg/ml) (Gibco, A38904-01) and laminin (2 μg/ml) (Merck, L2020). Cells were allowed to adhere for 2 to 3 h in a 5% CO_2_ environment at 37 °C before the addition of 2 ml of neurobasal A (Gibco) supplemented with 1× B27 (Gibco, 11530536), 1% P/S, and 0.5 mM GlutaMAX. At days 2 to 3 *in vitro*, cultures were treated with a combination of 100 μM anti-mitotic drugs including AraC, FUdR, and Uridine, to restrict the proliferation of dividing cells. DNA transfection using calcium phosphate (Promega, E1200) was carried out on cultures aged 7 days *in vitro* using a modified protocol previously described by Jiang and Chen (2006). In our method, we used a 50 μl of 2× HBS combined with a 50 μl solution containing H_2_O, DNA, and 5 μl of 2M CaCl_2_ to transfect one coverslip. Transfected neurons were either imaged or experimented 48 to 72 h post-transfection.

### Plasmids, Mutagenesis, and cDNA preparations

The cDNAs of wild-type (WT) human S1R and S1R E102Q cDNA with V5- and histidine-tag and S1R with EGFP-tag in pcDNA3.1(−) were used. The cDNA of porcine M2R, porcine M2R-CFP, M2R-EGFP, rat M4R, GABA_B_R and mGluR2 and GIRK1/2 were all subcloned into pcDNA3.1(−). Mutations in M2R and M4R were introduced using the PfuUltra II Fusion HS DNA Polymerase kit (Agilent Technologies) and verified by DNA sequencing. M2R-EGFP construct was made by incorporating the EGFP at the end of M2R WT using the PfuUltra II Fusion HS DNA Polymerase kit (Agilent Technologies), and was subcloned into NotI and EcoRI site of pcDNA3.1(−) and then verified by DNA sequencing. M2R construct with an extracellular HA tag and EGFP at the N-terminal (HA-EGFP-M2R), provided by Prof R. Zhang (Huazhong University of Science and Technology), has previously been described (26).

### Electrophysiological recording

In the whole cell patch clamp recording from HEK293T cells, data were acquired using a patch clamp amplifier (AXOPATCH 200B, Molecular Devices), a digital analog converter (Digidata 1440A, Molecular Devices), and pCLAMP 10.7 software (Molecular Devices). HEK293T cells were attached to PLL-coated glass for 4 to 6 h before recording and placed in the recording chamber, and membrane currents were recorded under a whole-cell patch clamp using a glass micropipette with an access resistance of 3 to 5 MΩ when filled with 140 mM KCl, 5 mM Na_2_-ATP, 3 mM EGTA, 0.1 mM CaCl_2_, 10 mM HEPES, 5 mM MgCl_2_ (PH 7.3 with KOH). 140K^+^ solution (140 mM KCl, 4 mM NaCl, 1 mM CaCl_2_, 0.3 mM MgCl_2,_ and 10 mM HEPES, pH 7.4 with KOH) or 140 Na^+^ solution (140 mM NaCl, 4 mM KCl, 0.3 mM MgCl_2_, 1 mM CaCl_2_ and 5 mM HEPES, pH 7.4 with NaOH) were used as an extracellular solution for recordings. All experiments were performed at 25 to 28 °C. Ligands, 50 μM OXO-M (Sigma-Aldrich, 033M4724V), 100 μM GABA (Tocris, 5B/82928), 200 μM Glutamate (Sigma-Aldrich, 30K0199) in 140 K^+^ were delivered and wash out by gravity flow using a multi-valve controller system (VC-8 VALVE CONTROLLAER, Warner Instruments).

### Co-immunoprecipitation

HEK293T cells were cultured in 60 mm dishes until cells reach 80 to 90% confluency and then were co-transfected with M2R-EGFP or its mutants and S1R-V5-His or its mutants using the Avalanche-Everyday Transfection Reagent (Avalanche- Everyday) following the protocol of the supplier. Cells were lysed after 48 h of incubation. Cell lysate was preincubated with G-Sepharose (Protein G Sepharose 4 Fast Flow, GE Healthcare) in a rotating wheel for 1 h at 4 °C and then centrifuged to remove the G-Sepharose and the supernatant was collected. Then, mouse monoclonal anti-EGFP antibody (Sigma-Aldrich, 2 μg) for immunoprecipitating M2R or mouse monoclonal anti-V5 tag antibody (Invitrogen, 2 μg) for immunoprecipitating M2R was added to the supernatant with G-Sepharose and incubated in a rotating wheel for 2 h at 4 °C. After 2 h of rotating incubation, samples were washed with a wash buffer and centrifuged 3 times repeatedly. The wash buffer was completely removed following the final centrifuge, and the sample buffer containing SDS was added to the tubes and incubated for 1 h at 37 °C. Proteins were finally analyzed by Western blot.

### Western blot analysis

Total protein lysate and IP samples were separated by SDS/PAGE (SuperSepAce, 10–20%, 13 or 17 wells, FUJIFILM) and blotted to PVDF membranes (Sigma-Aldrich) by using a wet transfer system. Membranes were blotted by Blocking One (Nacalai Tesque Inc) and then incubated with monoclonal rabbit anti-M2R antibody (Abcam, dilution 1:1000) or polyclonal rabbit anti-His tag antibody (Bethyl Laboratories, dilution 1:1000) diluted in Blocking One solution for 1 h at room temperature. Membranes were then washed by PBST 3 times, and samples were incubated with anti-rabbit secondary antibody conjugated with horseradish peroxidase (GE Healthcare, dilution 1:5000) to visualize and detect by chemiluminescence (ImmunoStar zeta kit, FUJIFILM).

### Immunostaining of HeLa and hippocampal neuronal cells

Both HeLa cells and hippocampal neurons were washed twice in prewarmed 1xPBS and then fixed in 4% PFA for 20 min, followed by three washes with 1xPBS. Cells were permeabilized with 0.2% TX-100 for 10 min and incubated for 30 min in a blocking solution containing 1% bovine serum albumin (BSA), 0.1% Tween 20, and 22.5 mg/ml glycine. The detection of the CFP tag was performed by the application of anti-GFP antibodies (ab13970) at a dilution of 1/2000 in 1% BSA in PBST (0.1% Tween 20 in PBS) overnight at 4 °C. Subsequently, cells were washed three times with PBS for 5 min each. Alexa Fluor 488 (ab150169) conjugated goat anti-chicken was used for secondary detection at a dilution of 1/1000 for 1 h at room temperature, followed by three washes with PBS, 5 min each. For confocal imaging, coverslips were mounted onto microscope slides in Fluoroshield (Sigma, F6057) and sealed with nail vanish.

### Quantification of cell surface expression of M2R in HeLa and HEK293T cells

The detection of cell surface HA-M2R was performed by incubating live HeLa and HEK293T cells for 1 h with anti-HA antibodies (Biolegend, 901,501) diluted in conditioned media to a final concentration of 5 μg/ml. A volume of 500 μl was added to each 16 mm coverslip in a 12-well plate, sufficient to cover the cells and thereby increase cell-antibody contact. Following antibody incubation, cells were washed twice with prewarmed 1× phosphate-buffered saline (PBS) and then fixed with prewarmed 4% paraformaldehyde. After fixation, cells were washed three times with PBS and maintained in PBS until the secondary antibody application. Alexa Fluor 647 (Molecular Probes, A21235) conjugated goat anti-mouse IgG was used for secondary detection. Cells were incubated with the secondary antibodies for 1 h at a final concentration of 4 μg/ml, followed by three washes with PBS. For imaging, coverslips were mounted onto microscope slides in Fluoroshield (Sigma, F6057).

Quantification of the surface expression in HeLa and HEK293T cells was analyzed using Image J. The background was subtracted and then an ROI was drawn immediately around the cell and the integrated density was measured per cell.

### Confocal imaging

Confocal imaging was carried out either on live cells (when imaging M2R-EGFP), or fixed cells. For live cell imaging, hippocampal neurons were incubated in HBSS and imaged at room temperature with a 63×/oil objective and a Leica SP8 confocal microscope. For fixed-cell imaging, HeLa cells were mounted onto a slide after immunostaining. The image settings were as follows: EGFP and Alex Fluor 488 were excited with a 488 nm Argon laser and emission light was collected between 500 to 550 nm. For S1R-mKate, excitation was with the 633 nm He/Ne laser, and emission was collected between 630 and 660 nm.

Cell surface expression of extracellular HA tagged-M2R by live cell staining was detected using a 63×/oil objective lens and a Leica SP8 confocal microscope (HeLa cells) or a 60×/oil objective lens and a Nikon A1RSi confocal microscope (HEK293T cells). The emission settings are as follows: EGFP was excited with a 488 nm Argon laser and emission light was detected between 500 to 550 nm. M-Kate was excited with a 561 nm DPSS laser and emission was captured between 610 and 630 nm. Alex Fluor 647 used to visualize the HA tag was excited with a 633 nm He/Ne laser and emission light was detected between 660 and 700 nm.

### Data analyses

In the patch-clamp experiments, all data were analyzed by Clampfit 10.7 (Molecular Devices) and Igor Pro 5.0 (WaveMetrics) and are shown as mean ± SD from n single HEK293T cells. The current record in 140 Na^+^ was subtracted to remove the endogenous current from HEK293T cells. The current amplitude of the GIRK1/2 channel was recorded at −120 mV before and after the application of the agonists to various G_i/o_ coupled receptors. The activation ratio of the GIRK1/2 channel by various agonists was calculated by the current amplitude in the presence and absence of the ligand after subtraction of endogenous current in 140 Na^+^ (I_GIRK agonist (+)_/I_GIRK agonist (−)_). An activation ratio of 1.0 means there was no change in the current amplitude after the application of agonists.

Analyses of electrophysiological experiments in HEK293T cells were performed with cell numbers of n ≥ 4 for each group. Statistical differences were evaluated using Student’s *t* test (unpaired).

Analyses of IHC experiments in HeLa cells were performed with cell numbers of n ≥ 359 for each group. Statistical differences between the three groups were evaluated by Kruskal-Wallis ANOVA followed by Dunn’s multiple comparisons tests and that of the two groups were by Student’s *t* test (unpaired). Those in HEK293T cells were performed with cell numbers of n ≥ 58 for each group. Statistical differences between three groups were evaluated by one-way ANOVA followed by Tukey’s multiple comparisons tests, and that of the two groups were by Student’s *t* test (unpaired).

The values of *p* < 0.05 were judged to be statistically significant.

## Data availability

All the data for this study are included in the article.

## Conflict of interest

The authors declare that they have no conflicts of interest with the contents of this article.

## References

[1] Chu U.B., Ruoho A.E. (2016). Biochemical Pharmacology of the sigma-1 receptor. Mol. Pharmacol..

[2] Su T.P., Su T.C., Nakamura Y., Tsai S.Y. (2016). The sigma-1 receptor as a pluripotent modulator in living systems. Trends Pharmacol. Sci..

[3] Dreser A., Vollrath J.T., Sechi A., Johann S., Roos A., Yamoah A. (2017). The ALS-linked E102Q mutation in Sigma receptor-1 leads to ER stress-mediated defects in protein homeostasis and dysregulation of RNA-binding proteins. Cell Death Differ..

[4] Gromek K.A., Suchy F.P., Meddaugh H.R., Wrobel R.L., LaPointe L.M., Chu U.B. (2014). The oligomeric states of the purified sigma-1 receptor are stabilized by ligands. J. Biol. Chem..

[5] Mishra A.K., Mavlyutov T., Singh D.R., Biener G., Yang J., Oliver J.A. (2015). The sigma-1 receptors are present in monomeric and oligomeric forms in living cells in the presence and absence of ligands. Biochem. J..

[6] Schmidt H.R., Zheng S., Gurpinar E., Koehl A., Manglik A., Kruse A.C. (2016). Crystal structure of the human sigma1 receptor. Nature.

[7] Sharma N., Patel C., Shenkman M., Kessel A., Ben-Tal N., Lederkremer G.Z. (2021). The Sigma-1 receptor is an ER-localized type II membrane protein. J. Biol. Chem..

[8] Fontanilla D., Hajipour A.R., Pal A., Chu U.B., Arbabian M., Ruoho A.E. (2008). Probing the steroid binding domain-like I (SBDLI) of the sigma-1 receptor binding site using N-substituted photoaffinity labels. Biochemistry.

[9] Ortega-Roldan J.L., Ossa F., Schnell J.R. (2013). Characterization of the human sigma-1 receptor chaperone domain structure and binding immunoglobulin protein (BiP) interactions. J. Biol. Chem..

[10] Pal A., Hajipour A.R., Fontanilla D., Ramachandran S., Chu U.B., Mavlyutov T. (2007). Identification of regions of the sigma-1 receptor ligand binding site using a novel photoprobe. Mol. Pharmacol..

[11] Mavlyutov T.A., Epstein M.L., Andersen K.A., Ziskind-Conhaim L., Ruoho A.E. (2010). The sigma-1 receptor is enriched in postsynaptic sites of C-terminals in mouse motoneurons. An anatomical and behavioral study. Neuroscience.

[12] Ortiz-Renteria M., Juarez-Contreras R., Gonzalez-Ramirez R., Islas L.D., Sierra-Ramirez F., Llorente I. (2018). TRPV1 channels and the progesterone receptor Sig-1R interact to regulate pain. Proc. Natl. Acad. Sci. U. S. A..

[13] Hayashi T., Su T.P. (2007). Sigma-1 receptor chaperones at the ER-mitochondrion interface regulate Ca(2+) signaling and cell survival. Cell.

[14] Hayashi T., Kishida M., Nishizawa Y., Iijima M., Koriyama C., Nakamura M. (2012). Subcellular localization and putative role of VPS13A/chorein in dopaminergic neuronal cells. Biochem. Biophys. Res. Commun..

[15] Su T.P., Hayashi T., Maurice T., Buch S., Ruoho A.E. (2010). The sigma-1 receptor chaperone as an inter-organelle signaling modulator. Trends Pharmacol. Sci..

[16] Morales-Lazaro S.L., Gonzalez-Ramirez R., Rosenbaum T. (2019). Molecular interplay between the sigma-1 receptor, steroids, and ion channels. Front. Pharmacol..

[17] Kopanchuk S., Vavers E., Veiksina S., Ligi K., Zvejniece L., Dambrova M. (2022). Intracellular dynamics of the Sigma-1 receptor observed with super-resolution imaging microscopy. PLoS One.

[18] Sabeti J., Nelson T.E., Purdy R.H., Gruol D.L. (2007). Steroid pregnenolone sulfate enhances NMDA-receptor-independent long-term potentiation at hippocampal CA1 synapses: role for L-type calcium channels and sigma-receptors. Hippocampus.

[19] Srivats S., Balasuriya D., Pasche M., Vistal G., Edwardson J.M., Taylor C.W. (2016). Sigma1 receptors inhibit store-operated Ca2+ entry by attenuating coupling of STIM1 to Orai1. J. Cell Biol..

[20] Aydar E., Palmer C.P., Klyachko V.A., Jackson M.B. (2002). The sigma receptor as a ligand-regulated auxiliary potassium channel subunit. Neuron.

[21] Wickman K., Clapham D.E. (1995). Ion channel regulation by G proteins. Physiol. Rev..

[22] Karschin A. (1999). G protein regulation of inwardly rectifying K(+) channels. News Physiol. Sci..

[23] Logothetis D.E., Kurachi Y., Galper J., Neer E.J., Clapham D.E. (1987). The beta gamma subunits of GTP-binding proteins activate the muscarinic K+ channel in heart. Nature.

[24] Mistry R., Dowling M.R., Challiss R.A. (2005). An investigation of whether agonist-selective receptor conformations occur with respect to M2 and M4 muscarinic acetylcholine receptor signalling *via* Gi/o and Gs proteins. Br. J. Pharmacol..

[25] Fredriksson R., Lagerstrom M.C., Lundin L.G., Schioth H.B. (2003). The G-protein-coupled receptors in the human genome form five main families. Phylogenetic analysis, paralogon groups, and fingerprints. Mol. Pharmacol..

[26] Wan M., Zhang W., Tian Y., Xu C., Xu T., Liu J. (2015). Unraveling a molecular determinant for clathrin-independent internalization of the M2 muscarinic acetylcholine receptor. Sci. Rep..

[27] Bernard V., Normand E., Bloch B. (1992). Phenotypical characterization of the rat striatal neurons expressing muscarinic receptor genes. J. Neurosci..

[28] James M.K., Cubeddu L.X. (1987). Pharmacologic characterization and functional role of muscarinic autoreceptors in the rabbit striatum. J. Pharmacol. Exp. Ther..

[29] Ousman S.S., Frederick A., Lim E.F. (2017). Chaperone proteins in the central nervous system and peripheral nervous system after nerve injury. Front. Neurosci..

[30] Abramyan A.M., Yano H., Xu M., Liu L., Naing S., Fant A.D. (2020). The Glu102 mutation disrupts higher-order oligomerization of the sigma 1 receptor. Comput. Struct. Biotechnol. J..

[31] Hong W.C. (2020). Distinct regulation of sigma (1) receptor multimerization by its agonists and antagonists in transfected cells and rat liver membranes. J. Pharmacol. Exp. Ther..

[32] Smith S.B. (2017). Introduction to sigma receptors: their role in disease and as therapeutic targets. Adv. Exp. Med. Biol..

[33] Marcotti A., Fernandez-Trillo J., Gonzalez A., Vizcaino-Escoto M., Ros-Arlanzon P., Romero L. (2023). TRPA1 modulation by Sigma-1 receptor prevents oxaliplatin-induced painful peripheral neuropathy. Brain.

[34] Hasselmo M.E. (2006). The role of acetylcholine in learning and memory. Curr. Opin. Neurobiol..

[35] Schliebs R., Arendt T. (2011). The cholinergic system in aging and neuronal degeneration. Behav. Brain Res..

[36] Krapivinsky G., Gordon E.A., Wickman K., Velimirovic B., Krapivinsky L., Clapham D.E. (1995). The G-protein-gated atrial K+ channel IKACh is a heteromultimer of two inwardly rectifying K(+)-channel proteins. Nature.

[37] Bymaster F.P., Carter P.A., Zhang L., Falcone J.F., Stengel P.W., Cohen M.L. (2001). Investigations into the physiological role of muscarinic M2 and M4 muscarinic and M4 receptor subtypes using receptor knockout mice. Life Sci..

[38] Abdullah C.S., Alam S., Aishwarya R., Miriyala S., Panchatcharam M., Bhuiyan M.A.N. (2018). Cardiac dysfunction in the sigma 1 receptor knockout mouse associated with impaired mitochondrial dynamics and bioenergetics. J. Am. Heart Assoc..

[39] Ela C., Barg J., Vogel Z., Hasin Y., Eilam Y. (1994). Sigma receptor ligands modulate contractility, Ca++ influx and beating rate in cultured cardiac myocytes. J. Pharmacol. Exp. Ther..

